# pH-Dependent Assembly and Segregation of the Coiled-Coil Segments of Yeast Putative Cargo Receptors Emp46p and Emp47p

**DOI:** 10.1371/journal.pone.0140287

**Published:** 2015-10-08

**Authors:** Kentaro Ishii, Hiroki Enda, Masanori Noda, Megumi Kajino, Akemi Kim, Eiji Kurimoto, Ken Sato, Akihiko Nakano, Yuji Kobayashi, Hirokazu Yagi, Susumu Uchiyama, Koichi Kato

**Affiliations:** 1 Okazaki Institute for Integrative Bioscience, National Institutes of Natural Sciences, Okazaki, Aichi, Japan; 2 Graduate School and Faculty of Pharmaceutical Sciences, Nagoya City University, Nagoya, Aichi, Japan; 3 Department of Biotechnology, Graduate School of Engineering, Osaka University, Suita, Osaka, Japan; 4 Faculty of Pharmacy, Meijo University, Nagoya, Aichi, Japan; 5 Department of Life Sciences, Graduate School of Arts and Sciences, The University of Tokyo, Meguro-ku, Tokyo, Japan; 6 Department of Biological Sciences, Graduate School of Science, The University of Tokyo, Bunkyo-ku, Tokyo, Japan; 7 Live Cell Super-Resolution Imaging Research Team, RIKEN Center for Advanced Photonics, Wako, Saitama, Japan; 8 Institute for Molecular Science, National Institutes of Natural Sciences, Okazaki, Aichi, Japan; Consejo Superior de Investigaciones Cientificas, SPAIN

## Abstract

Emp46p and Emp47p are yeast putative cargo receptors that recycle between the endoplasmic reticulum and the Golgi apparatus. These receptors can form complexes in a pH-dependent manner, but their molecular mechanisms remain unclear. Here, we successfully reproduced their interactions *in vitro* solely with their coiled-coil segments, which form stable heterotetramers in the neutral condition but segregate at lower pH. Mutational data identified a key glutamate residue of Emp46p that serves as the pH-sensing switch of their oligomer formation. Our findings elucidate the mechanisms of the dynamic cargo receptor interactions in the secretory pathway and the design framework of the environment-responsive molecular assembly and disassembly systems.

## Introduction

Eukaryotic cells are equipped with a vesicular transport system for sorting and translocating cargo proteins in the secretory pathway. Protein transport in the early secretory pathway is mediated by COPII-coated vesicles, which bud from the endoplasmic reticulum (ER) and travel to the Golgi compartment [1]. Growing evidence indicates that several soluble secretory proteins require specific transmembrane cargo receptors as adaptors between these cargos and the COPII coats for selective packaging [2, 3].

In mammalian cells, ERGIC-53 has been identified as a cargo receptor that recycles between the ER and the Golgi apparatus and contributes to the anterograde transport of several glycoproteins, including the lysosomal glycoproteins cathepsin C and cathepsin Z and blood coagulation factors V and VIII [3]. ERGIC-53 and its homolog VIP36 are type I membrane proteins harboring a carbohydrate recognition domain (CRD), which structurally resembles leguminous lectins, such as concanavalin A, and binds high-mannose-type oligosaccharides that are displayed on the cargos in Ca^2+^- and pH-dependent manners [3–6]. In ERGIC-53, CRD is linked to a coiled-coil segment that is followed by a transmembrane region with a C-terminal cytoplasmic tail containing both ER exit and retrieval signals. Thus, ERGIC-53 forms a disulfide-linked homodimer or homohexamer through the coiled-coil segment and specifically interacts, through the CRD domain, with a Ca^2+^-binding protein MCFD2, which cooperates with ERGIC-53 in the vesicular transport of blood coagulation factors V and VIII [7].

Emp46p and Emp47p, the yeast homologs of ERGIC-53, are considered putative cargo receptors recycling the ER and the Golgi apparatus because disruption of both proteins significantly impairs the secretion of a subset of glycoproteins, although their client cargos remain unidentified as yet [8–11]. Like ERGIC-53, these yeast proteins share homologous CRDs and possess a putative coiled-coil segment followed by a transmembrane region with a C-terminal tail. Despite their structural homology with ERGIC-53, their CRDs are independent of Ca^2+^ and their coiled-coil segments contain no cysteine residue. Immunoprecipitation of differently tagged recombinant proteins expressed in the *emp46*Δ*emp47*Δ cells indicated that, in the ER, Emp46p and Emp47p are monomeric and homo-oligomeric, respectively, and capable of interacting with each other [9]. These interactions are mediated through their coiled-coil segments. Emp47p is required for the ER exit of Emp46p; however, the converse is not true. Emp46–Emp47 interaction was not detected in the Golgi fraction. On the basis of these findings, it has been suggested that Emp47p is a receptor for Emp46p, escorting it from the ER to the Golgi apparatus. However, molecular mechanisms of the assembly and disassembly of these proteins are largely unknown. Therefore, we herein characterize the putative interactions of the coiled-coil segments of Emp46p and Emp47p to provide a molecular basis of the dynamic processes involved in vesicular transport.

## Results and Discussion

### Homo-oligomeric states of Emp46p^CC^ and Emp47p^CC^

**Fig 1:**
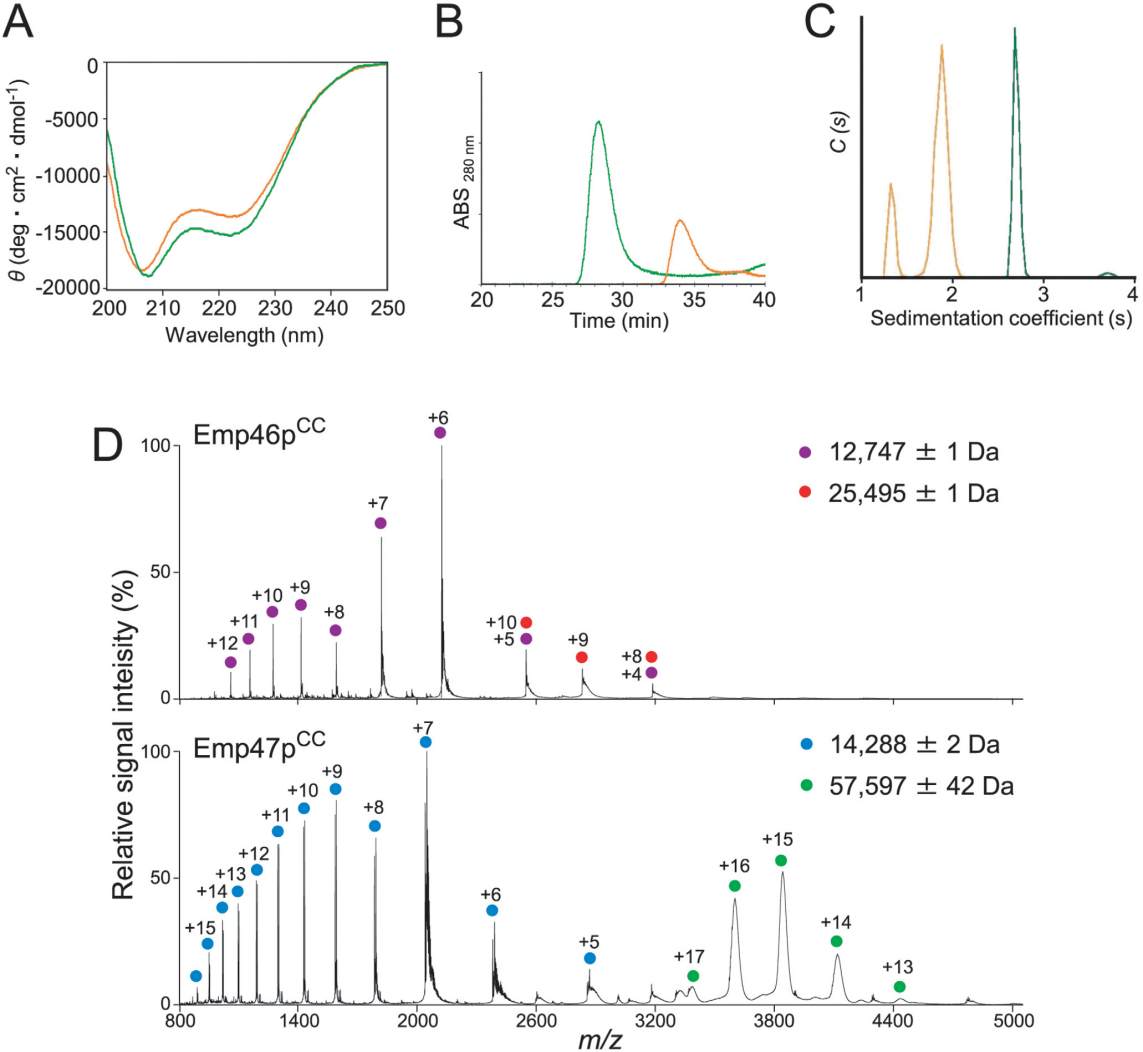
Characterization of oligomeric states of Emp46p^CC^ and Emp47p^CC^. (A) CD spectra of Emp46p^CC^ (orange) and Emp47p^CC^ (green). (B) Gel filtration profiles of Emp46p^CC^ (orange) and Emp47p^CC^ (green) at pH 7.4. (C) Distribution of sedimentation coefficients derived from sedimentation velocity analytical ultracentrifugation (SV-AUC) experiments. The orange line indicates Emp46p^CC^ (75 μM), whereas the green line indicates Emp47p^CC^ (75 μM). (D) Mass spectra of Emp46p^CC^ (top) and Emp47p^CC^ (bottom) at pH 7.4. Peaks corresponding to the monomer and dimer of Emp46p^CC^ are indicated by purple and red dots with charge states, respectively. Peaks corresponding to the monomer and tetramer of Emp47p^CC^ are indicated by blue and green dots with charge states, respectively.

We attempted to reproduce oligomer formation of Emp46p and Emp47p using their coiled-coil segments. The bacterially expressed coiled-coil segment of Emp46p^CC^ (252–355) and Emp47p^CC^ (271–274) were subjected to circular dichroism (CD) spectroscopic analysis, which confirmed the α-helical structures of both recombinant proteins (Fig 1A). The oligomeric states of Emp46p^CC^ and Emp47p^CC^ were subsequently characterized by biochemical and biophysical methods. Gel filtration and chemical cross-linking experiments indicated that Emp47p^CC^, but not Emp46p^CC^, formed a homotetramer (Fig 1B and S1 Fig). For more accurate determination of oligomeric states, Emp46p^CC^ and Emp47p^CC^ were subjected to sedimentation velocity analytical ultracentrifugation (SV-AUC) and mass spectroscopy (MS) analyses. SV-AUC data showed that Emp46p^CC^ exhibited two species, with peaks at 1.2 S and 1.9 S, from which their molecular weight (M_w_) were estimated to be 12.7 and 21.2 kDa, respectively (Fig 1C). The populations of the two peaks for Emp46p^CC^ were concentration dependent (S2A Fig) and the s-value of the species with the higher s-value changed as the concentration changed. This is a typical result for an association—dissociation system with rapid dissociation kinetics [12]. The MS analysis that consistently provided accurate molecular masses of the two species, both monomer and dimer, were detected for Emp46p^CC^ (Fig 1D). Meanwhile, Emp47p^CC^ had a single peak at approximately 2.7 S with apparently no concentration dependence, providing an M_w_ of 63.3 kDa (Fig 1C and S2B Fig). These data revealed that Emp46p^CC^ underwent monomer—dimer equilibrium, whereas Emp47p^CC^ formed a stable tetramer, giving quantitative and updated views from the previous immunoprecipitation analysis [9].

### Hetero-oligomeric coiled-coil interactions between Emp46p^CC^ and Emp47p^CC^

**Fig 2:**
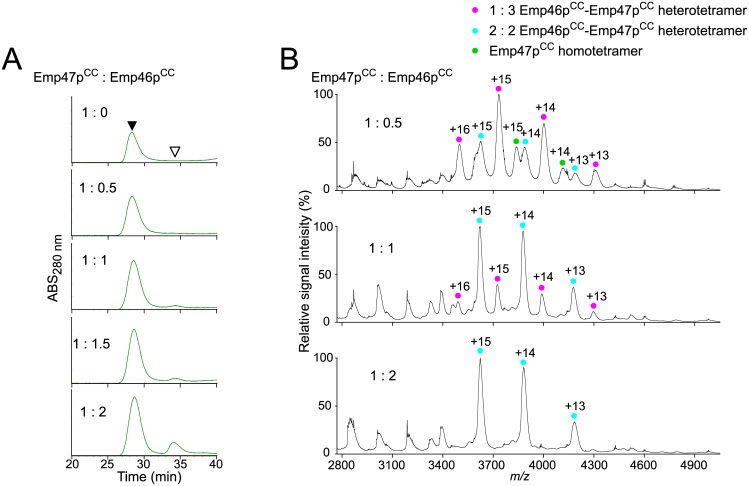
Characterization of complex formation between Emp46p^CC^ and Emp47p^CC^ at pH 7.4. (A) Gel-filtration profiles of the mixture of Emp46p^CC^ and Emp47p^CC^ at 1:0, 1:0.5, 1:1, 1:1.5, and 1:2 molar ratios. White and black arrows indicate the elution positions of Emp46p^CC^ alone and oligomers involving Emp47p^CC^ (and Emp46p^CC^), respectively. (B) Mass spectra of the mixtures of Emp46p^CC^ and Emp47p^CC^ at pH 7.4. The concentration of Emp47p^CC^ was fixed and that of Emp46p^CC^ was varied at the molar ratio indicated. Peaks corresponding to 1:3 and 2:2 heterotetramers of Emp46p^CC^ and Emp47p^CC^ are indicated by magenta and cyan dots with charge states, respectively. Peaks corresponding to the homotetramer of Emp47p^CC^ are indicated by a green dot with charge states.

Next, we attempt to evaluate the potential interaction between Emp46p^CC^ and Emp47p^CC^. In gel filtration of Emp47p^CC^ titrated with Emp46p^CC^ under a neutral condition, that is, pH 7.4, less than one molar equivalent of Emp46p^CC^ co-eluted with Emp47p^CC^, indicating the formation of hetero-oligomers with Emp47p^CC^ (Fig 2A). Chemical cross-linking experiments detected a covalently linked heterodimer on SDS-PAGE (S1 Fig). MS data demonstrated that Emp46p^CC^ and Emp47p^CC^ formed heterotetramers with a 1:3 or 2:2 stoichiometry; the later increased with increasing quantities of Emp46p^CC^ (Fig 2B). All of these data indicate that the homotypic and heterotypic interactions involving Emp46p and Emp47p, which were previously observed by immunoprecipitation, are well reproduced *in vitro* solely with their coiled-coil segments and characterized more quantitatively.

**Fig 3:**
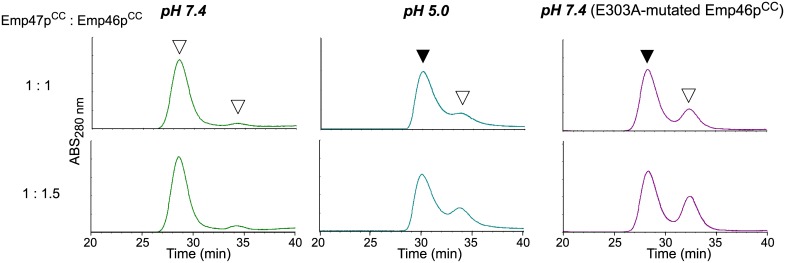
Gel filtration profiles of the mixture of Emp46p^CC^ and Emp47p^CC^ under pH 7.4 (left) and 5.0 (center) and the mixture of Emp46p^CC^ mutant E303A and Emp47p^CC^ under pH 7.4 (right) at 1:1 and 1:1.5 molar ratios. White arrows indicate the elution positions of Emp46p^CC^ or its E303A mutant. Black arrows indicate the elution positions of the hetero-oligomers involving Emp47p^CC^.

In mass spectra measured at pH 5.0, the homo-oligomeric and hetero-oligomeric forms of the Emp46p^CC^ and Emp47p^CC^ were detected with the identical stoichiometric numbers with those observed at pH 7.0 (S3 and S4 Figs). However, the quantitative comparison of the MS data was difficult in terms of relative incidence of the complex species because relative ionization efficiencies of protein complexes are generally different under different pH conditions. For quantitative estimation of the relative population of the complexes, we employed gel filtration. The results clearly demonstrated that the Emp46p^CC^–Emp47p^CC^ heterotetramer formation was impaired at pH 5.0 (Fig 3). Emp46p and Emp47p have been reported to form complexes in the ER but segregate in the Golgi compartment. H^+^ pumping by v-ATPase causes progressive acidification from the ER through the Golgi to the *trans*-Golgi network with typical pH values of ER, *cis*-Golgi, and *trans*-Golgi network that are reported to be 7.2, 6.4, and 5.4, respectively. The present data demonstrate that the Emp46p^CC^–Emp47^CC^ interaction is weakened by lowering the solution pH. Based on these findings, we suggest that assembly and segregation of these cargo receptors during their transit from the ER to the Golgi compartment are at least in part ascribed to their pH-dependent coiled-coil interaction.

### Key glutamate of pH-dependent segregation of Emp46p^CC^ and Emp47p^CC^

**Fig 4:**
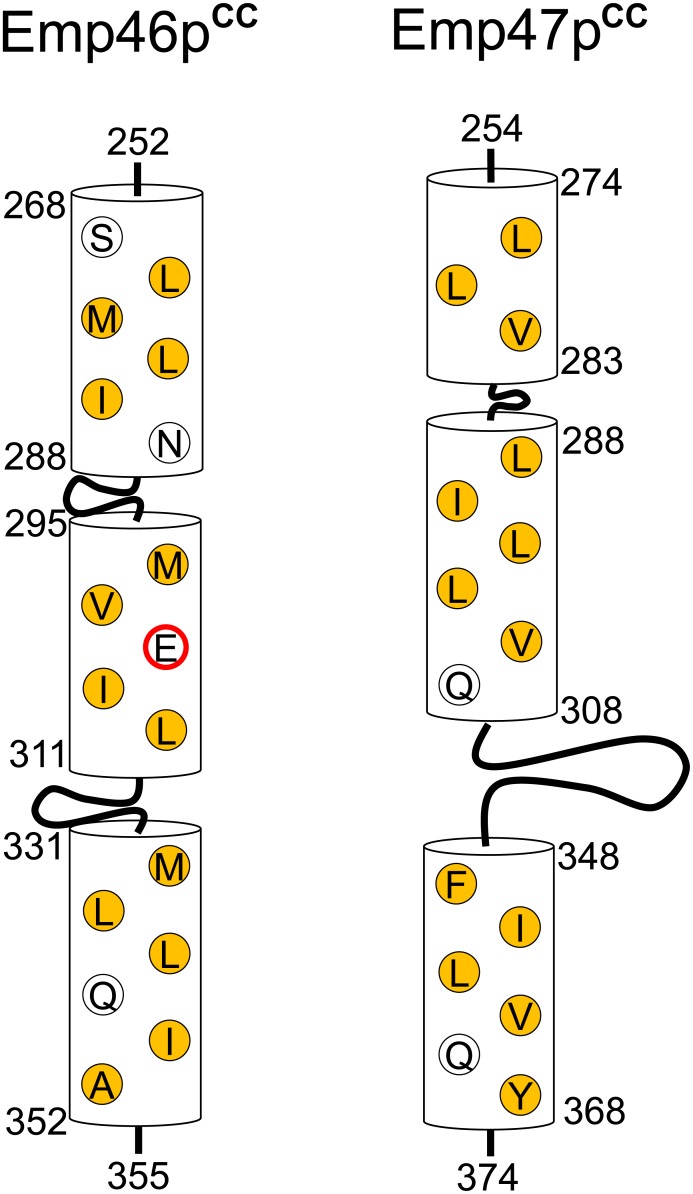
Schematic of the coiled-coil segments of Emp46p^CC^ and Emp47p^CC^. Residues consisting of a hydrophobic core are represented. Hydrophobic residues are represented by yellow, whereas Glu303 is represented with a red circle.

In general, coiled-coil segments exhibit amphiphilic α-helices, which are characterized by the heptad repeat, *HPPHPPP*, of hydrophobic (*H*) and polar (*H*) residues, and are assembled to bury their hydrophobic *H*/*H* surface. A glutamate residue (Glu303) is located in the hydrophobic surface of Emp46p^CC^ (Fig 4). It is possible that this glutamate negatively contributes to the homo-oligomer formation of Emp46p^CC^ because of the potential electrostatic repulsion caused by its negative charge under neutral conditions; however, this might be preferably accommodated in the tetrameric complex formed with Emp47p^CC^. On the basis of this idea, we examined the possible effect of the alanine substitution of Glu303 in Emp46p^CC^ on its oligomer formation. The E303A mutant of Emp46p^CC^ maintained the integrity of the α-helical structure (S5A Fig) and tended to form a homodimer in comparison with the wild type (S5B Fig). This mutation impaired the Emp46p^CC^–Emp47p^CC^ interaction even at pH 7.4 as shown by gel filtration (Fig 3) and chemical cross-linking analysis (S5B Fig). Consistently, in native mass spectra, this mutant was detected only as the 1:3 Emp46p^CC^/Emp47p^CC^ complex even under the condition where the wild-type Emp46p^CC^ was observed exclusively as the 2:2 complex with Emp47p^CC^ (S6 Fig). These results indicate that Glu303 of Emp46p^CC^ serves as the pH-sensing switch of assembly and segregation of Emp46p and Emp47p. The pH-dependent coiled-coil interaction is exemplified by macrophage scavenger receptor, in which the trimeric α-helical coiled-coil domain exhibits conformational change depending on solution pH [13]. In this system, the C-terminal coiled-coil segment forms a random structure at neutral pH and a coiled-coil structure under acidic condition. Moreover, it has been reported that a glutamate residue located in the hydrophobic surface of this coiled-coil segment plays a critical role in this pH-dependent conformational transition [13]. Our results along with these data suggest possible mechanistic similarities shared among the pH-dependent coiled-coil interactions.

In summary, the present study successfully characterized the pH-dependent coiled-coil interactions of Emp46p and Emp47p, identifying the key residue that controls this interaction. These results contribute toward understanding the molecular mechanisms underlying the dynamic cargo receptor interactions in the yeast secretory pathway. Moreover, our findings will provide a framework for designing molecular assembly and disassembly systems mediated by coiled-coil interactions responsive to changes in environmental conditions.

## Materials and Methods

### Protein expression and purification

The coiled-coil segments of Emp46p and Emp47p were identified using COILS/PCOILS (http://toolkit.tuebingen.mpg.de/pcoils) [14]. Plasmid vectors that contained the genes of the coiled-coil segment of Emp46p^CC^ (252–355) or Emp47p^CC^ (254–374) produced by amplification of polymerase chain reaction were constructed and cloned for their expression as a fusion protein with hexahistidine-tagged ubiquitin (His_6_-Ub) using a pET28a(+) vector (Novagene) or a His_6_-tagged protein using a pET28b(+) vector (Novagene). These vectors were transformed into the *Escherichia coli* strain BL21-CodonPlus. The cells were grown at 37°C in Luria—Bertani medium containing 15 μg/mL of kanamycin. Protein expression was induced by adding 0.5 mM isopropyl-β_D_-thiogalactopyranoside when absorbance reached 0.8 at 600 nm. After 3 h, cells were harvested, suspended in 50 mM Tris—HCl (pH 8.0), and subsequently, disrupted by sonication. After centrifugation, the His_6_-Ub-tagged or His_6_-tagged proteins were purified in a Chelating Sepharose Fast Flow column (GE Healthcare Life Sciences). The Emp46p^CC^ and Emp47p^CC^ were enzymatically cleaved from the His_6_–Ub moiety using recombinant glutathione *S*-transferase-tagged yeast ubiquitin hydrolase-1, as described previously [15], or cleaved from the His_6_-segment using thrombin protease (GE Healthcare), and then purified by chromatography using a gel filtration column (Superose 12 column 10/300 GL, GE Healthcare).

### Circular dichroism measurements

The purified Emp46p^CC^ and Emp47p^CC^ (0.1 mg/mL) were dissolved in 50 mM Tris—HCl buffer (pH 7.6) containing 0.15 M NaCl. Measurements of CD spectra were performed in a 1-mm quartz cuvette at room temperature using a spectropolarimeter (J-725, JASCO). After subtraction of the spectrum of the buffer alone, data were represented as mean residue ellipticities.

### Gel filtration

The purified Emp47p^CC^ protein (70 μM), either alone or in the presence of Emp46p^CC^ or its E303A mutants, was subjected to gel filtration using a Superose 12 column (GE Healthcare), equilibrated with 10 mM phosphate buffer (pH 7.4) containing 150 mM NaCl or 50 mM acetate buffer (pH 5.0) containing 150 mM NaCl.

### Chemical cross-linking

The purified Emp46p^CC^ (or its E303A mutant) and Emp47p^CC^ proteins were dialyzed against 100 mM carbonate/bicarbonate buffer (pH 8.9). Next, dimethyl suberimidate dihydrochloride (DMS) was added to derive a final concentration of 0.1 mg/mL and incubated for 60 or 120 min at room temperature. The cross-linking reaction was quenched by adding 500 mM sodium phosphate buffer (pH 6) to adjust pH.

### Analytical ultracentrifugation

SV-AUC experiments were performed in 10 mM mmol/L phosphate buffer (pH 7.4) containing 150 mM NaCl using a Proteomelab XL-I Analytical Ultracentrifuge (Beckman—Coulter). The samples of Emp46p^CC^ (75 μM), Emp47p^CC^ (75 μM), and their mixtures at varying protein concentrations were measured. Runs were conducted at 60,000 rpm, at a temperature of 20°C using 12-mm aluminum double-sector centerpieces and a four-hole An60 Ti analytical rotor that was equilibrated to 20°C. The sedimentating boundaries were monitored using Rayleigh interferometric detection at 655 nm. In total, 150 scans were obtained between 5.9 cm and 7.25 cm from the center of the rotation axis. All SV-AUC raw data were analyzed using the continuous *C* (s) distribution model provided by the SEDFIT11.71 software program. The parameters used for the analysis, that is, the partial specific volume of the protein calculated from amino acid composition (Emp46p^CC^: 0.73710 cm^3^/g, Emp47p^CC^: 0.73835 cm^3^/g), buffer density (*ρ* = 1.00530 g/ cm^3^), and buffer viscosity (*η* = 1.0193 cP) were estimated using SEDNTERP ver.1.09 [16].

### Mass spectrometry under non-denaturing conditions

The purified Emp46p^CC^ (or its E303A mutant) and Emp47p^CC^ proteins (100 μM) were buffer-exchanged into 200 mM ammonium acetate at pH 7.4 by passing through a Bio-Spin 6 column (Bio-Rad). The samples (43 μM) were immediately analyzed by nanoflow electrospray using in-house made gold-coated glass capillaries (approximately 2 μL sample loaded per analysis). The mixtures of Emp47p^CC^ (33 μM) and 17, 33, and 67 μM Emp46p^CC^ were incubated at 25°C for 30 min, and analyzed by electron spray ionization MS after the buffer exchange. Spectra were recorded on a SYNAPT G2-S*i* HDMS mass spectrometer (Waters, Manchester, UK) in positive ionization mode at 1.63 kV with 150 V sampling cone voltage and source offset voltage, 0 V trap and transfer collision energy, and 5 mL/min trap gas flow. The spectra were calibrated using 1 mg/mL cesium iodide and analyzed by Mass Lynx software (Waters).

## Supporting Information

S1 Fig Detection of oligomeric states of Emp46p^CC^ *and* Emp47p^CC^ by chemical cross lining analysis. Emp46p^CC^, Emp47p^CC^, and their mixture of Emp46p^CC^ and Emp47p^CC^ were individually incubated with DMS for 60 min. The reaction mixtures were analyzed using SDS-PAGE.
(PDF)

S2 Fig Sedimentation coefficient distributions of Emp46p^CC^ and Emp47p^CC^ at different concentrations. (A) Emp46p^CC^. (B) Emp47p^CC^. Solid line: 150 μM, dashed line: 75 μM, dotted line: 15 μM.
(PDF)

S3 Fig Mass spectra of Emp46p^CC^ and Emp47p^CC^ at pH 5.0. Emp46p^CC^ (top). Emp47p^CC^ (bottom). Peaks corresponding to the monomer and dimer of Emp46p^CC^ are indicated by purple and red dots with charge states, respectively. Peaks corresponding to the monomer, trimer, and tetramer of Emp47p^CC^ are indicated by blue, yellow, and green dots with charge states, respectively.
(PDF)

S4 Fig Mass spectra of the mixtures of Emp46p^CC^ and Emp47p^CC^ at pH 5.0. The concentration of Emp47p^CC^ was fixed and that of Emp46p^CC^ was varied at the molar ratios indicated. Peaks corresponding to 1:3 and 2:2 heterotetramers of Emp46p^CC^ and Emp47p^CC^ are indicated by magenta and cyan dots with charge states, respectively. Peaks corresponding to the homotetramer of Emp47p^CC^ are indicated by a green dot with charge states.
(PDF)

S5 Fig Characterization of conformation and oligomeric states of Emp46p^CC^ mutant E303A. (A) CD spectra of the wild-type (left) and E303A-mutated Emp46p^CC^ (right). (B) DMS cross-linking of the wild-type and E303A-mutated Emp46p^CC^ in the presence or absence of Emp47p^CC^. The proteins and their mixtures were individually incubated with DMS for 60 min. The reaction mixtures were analyzed using SDS-PAGE.
(PDF)

S6 Fig Mass spectra of the mixtures of Emp47p^CC^ and the E303-mutated Emp46p^CC^ at pH 7.4. The concentration of Emp47p^CC^ was fixed and that of the mutated Emp46p^CC^ was varied at the molar ratios indicated. Peaks corresponding to 1:3 heterotetramers of Emp47p^CC^ and the E303A-mutated Emp46p^CC^ are indicated by magenta dots with charge states. Peaks corresponding to the homotetramer of Emp47p^CC^ are indicated by a green dot with charge states.
(PDF)
