# Supplementary material for: pH-Dependent Assembly and Segregation of the Coiled-Coil Segments of Yeast Putative Cargo Receptors Emp46p and Emp47p
Source: PLoS One. 2015 Oct 8;10(10):e0140287. doi: 10.1371/journal.pone.0140287 (PMC4598028; doi:10.1371/journal.pone.0140287)
Supplement: S1 Fig — Emp46pCC, Emp47pCC, and their mixture of Emp46pCC and Emp47pCC were individually incubated with DMS for 60 min. The reaction mixtures were analyzed using SDS-PAGE. (PDF) [file pone.0140287.s001.pdf]

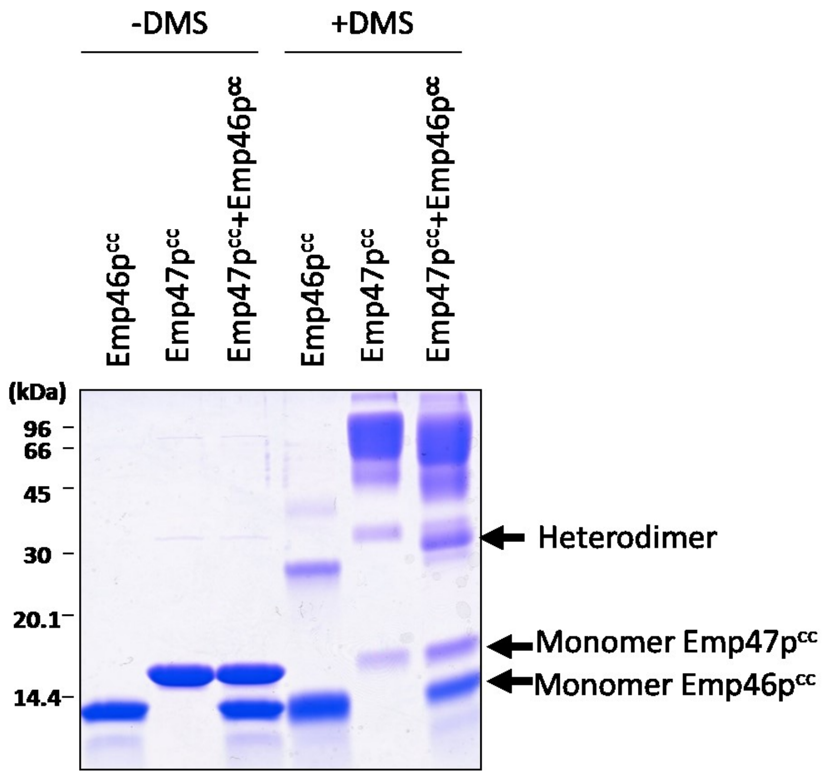

S1 Fig. Detection of oligomeric states of Emp46p^{CC} and Emp47p^{CC} by chemical cross lining analysis. Emp46p^{CC}, Emp47p^{CC}, and their mixture of Emp46p^{CC} and Emp47p^{CC} were individually incubated with DMS for 60 min. The reaction mixtures were analyzed using SDS-PAGE.
