# Supplementary material for: pH-Dependent Assembly and Segregation of the Coiled-Coil Segments of Yeast Putative Cargo Receptors Emp46p and Emp47p
Source: PLoS One. 2015 Oct 8;10(10):e0140287. doi: 10.1371/journal.pone.0140287 (PMC4598028; doi:10.1371/journal.pone.0140287)
Supplement: S2 Fig — (A) Emp46pCC. (B) Emp47pCC. Solid line: 150 μM, dashed line: 75 μM, dotted line: 15 μM. (PDF) [file pone.0140287.s002.pdf]

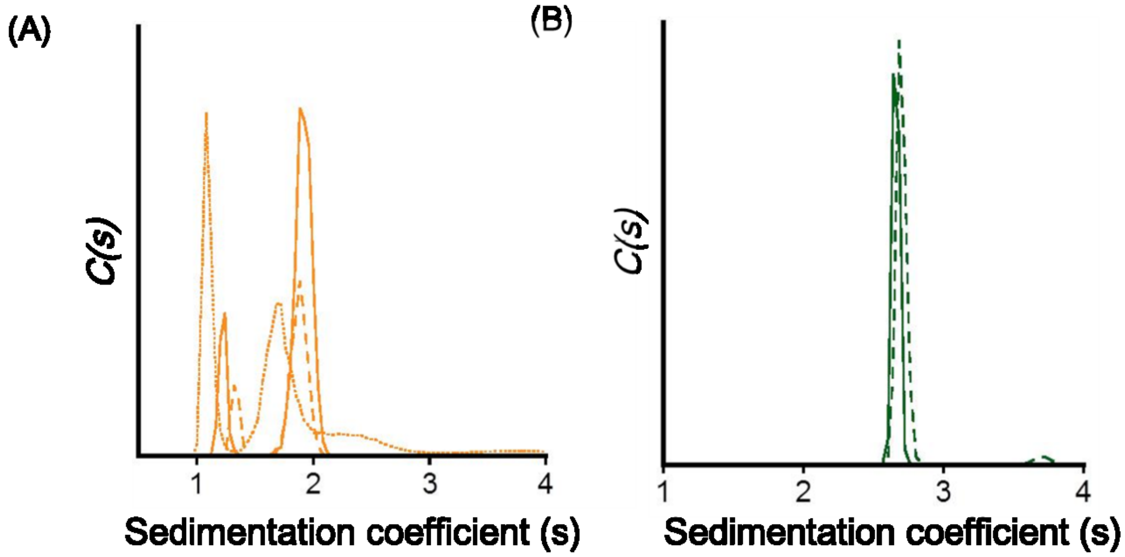

S2 Fig. Sedimentation coefficient distributions of Emp46p^{CC} and Emp47p^{CC} at different concentrations. (A) Emp46p^{CC}. (B) Emp47p^{CC}. Solid line: 150 µM, dashed line: 75 µM, dotted line: 15 µM.
