# Supplementary material for: pH-Dependent Assembly and Segregation of the Coiled-Coil Segments of Yeast Putative Cargo Receptors Emp46p and Emp47p
Source: PLoS One. 2015 Oct 8;10(10):e0140287. doi: 10.1371/journal.pone.0140287 (PMC4598028; doi:10.1371/journal.pone.0140287)
Supplement: S3 Fig — Emp46pCC (top). Emp47pCC (bottom). Peaks corresponding to the monomer and dimer of Emp46pCC are indicated by purple and red dots with charge states, respectively. Peaks corresponding to the monomer, trimer, and tetramer of Emp47pCC are indicated by blue, yellow, and green dots with charge states, respectively. (PDF) [file pone.0140287.s003.pdf]

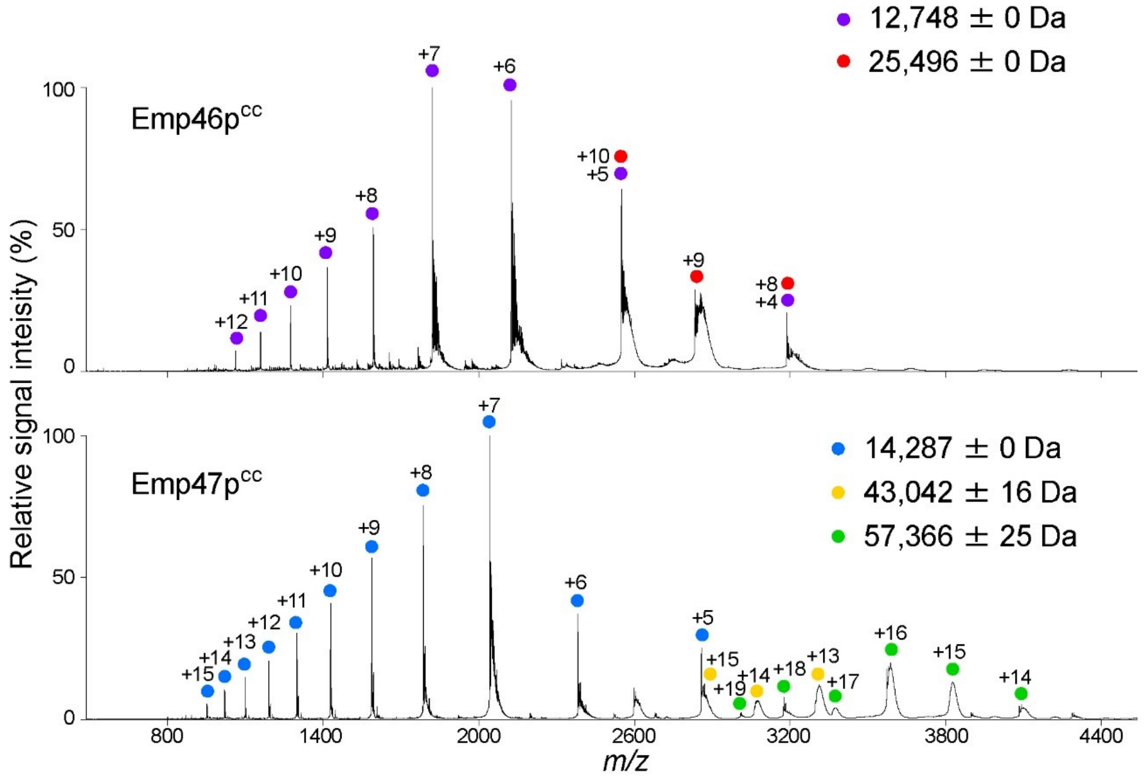

S3 Fig. Mass spectra of Emp46p^{CC} and Emp47p^{CC} at pH 5.0. Emp46p^{CC} (top). Emp47p^{CC} (bottom). Peaks corresponding to the monomer and dimer of Emp46p^{CC} are indicated by purple and red dots with charge states, respectively. Peaks corresponding to the monomer, trimer, and tetramer of Emp47p^{CC} are indicated by blue, yellow, and green dots with charge states, respectively.
