# Supplementary material for: pH-Dependent Assembly and Segregation of the Coiled-Coil Segments of Yeast Putative Cargo Receptors Emp46p and Emp47p
Source: PLoS One. 2015 Oct 8;10(10):e0140287. doi: 10.1371/journal.pone.0140287 (PMC4598028; doi:10.1371/journal.pone.0140287)
Supplement: S5 Fig — (A) CD spectra of the wild-type (left) and E303A-mutated Emp46pCC (right). (B) DMS cross-linking of the wild-type and E303A-mutated Emp46pCC in the presence or absence of Emp47pCC. The proteins and their mixtures were individually incubated with DMS for 60 min. The reaction mixtures were analyzed using SDS-PAGE. (PDF) [file pone.0140287.s005.pdf]

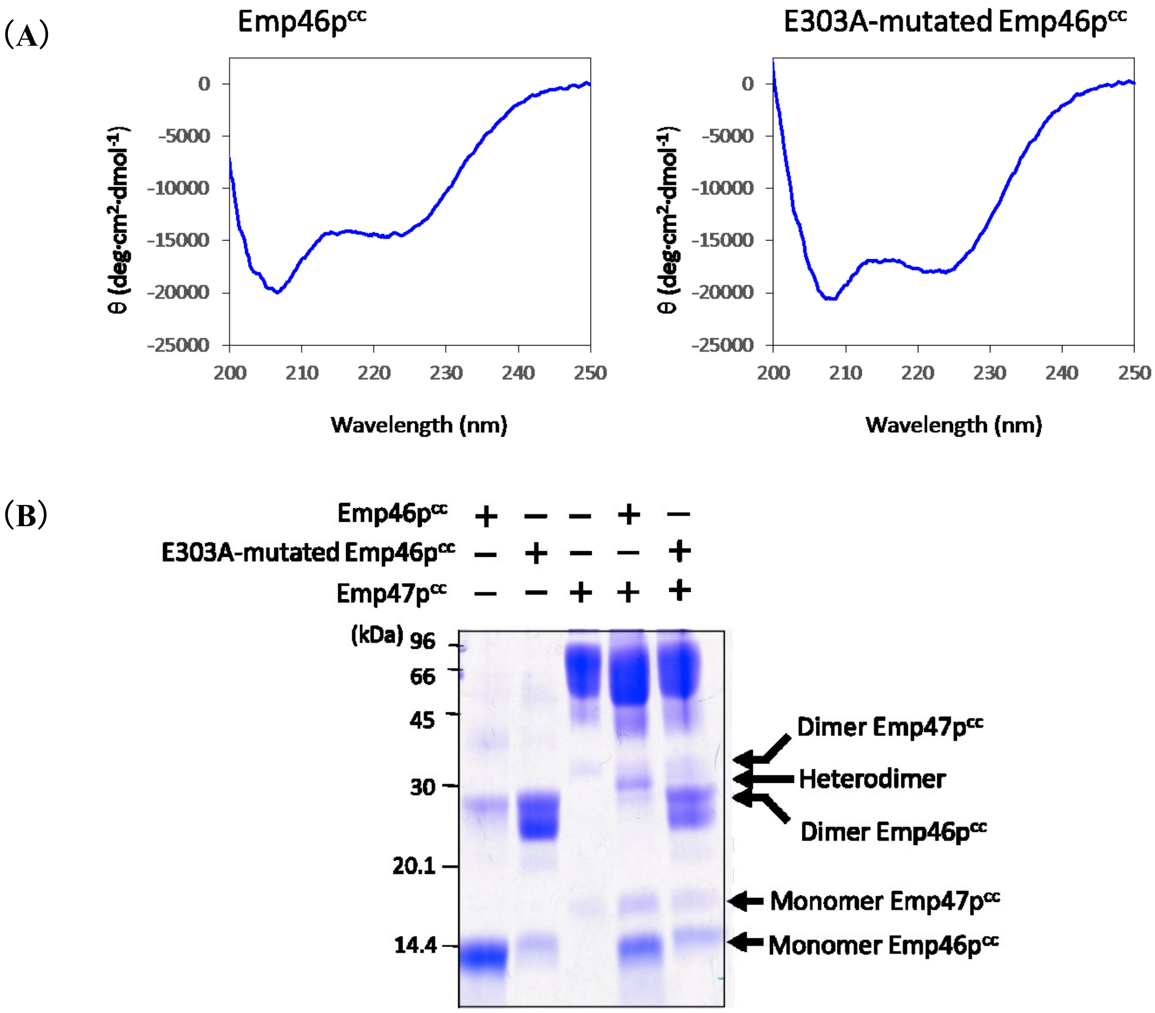

S5 Fig. Characterization of conformation and oligomeric states of E303A-mutated Emp46p^{CC}. (A) CD spectra of the wild-type (left) and E303A-mutated Emp46p^{CC} (right). (B) DMS cross-linking of the wild-type and E303A-mutated Emp46p^{CC} in the presence or absence of Emp47p^{CC}. The proteins and their mixtures were individually incubated with DMS for 120 min. The reaction mixtures were analyzed using SDS-PAGE.
