# Supplementary material for: pH-Dependent Assembly and Segregation of the Coiled-Coil Segments of Yeast Putative Cargo Receptors Emp46p and Emp47p
Source: PLoS One. 2015 Oct 8;10(10):e0140287. doi: 10.1371/journal.pone.0140287 (PMC4598028; doi:10.1371/journal.pone.0140287)
Supplement: S6 Fig — The concentration of Emp47pCC was fixed and that of the mutated Emp46pCC was varied at the molar ratios indicated. Peaks corresponding to 1:3 heterotetramers of Emp47pCC and the E303A-mutated Emp46pCC are indicated by magenta dots with charge states. Peaks corresponding to the homotetramer of Emp47pCC are indicated by a green dot with charge states. (PDF) [file pone.0140287.s006.pdf]

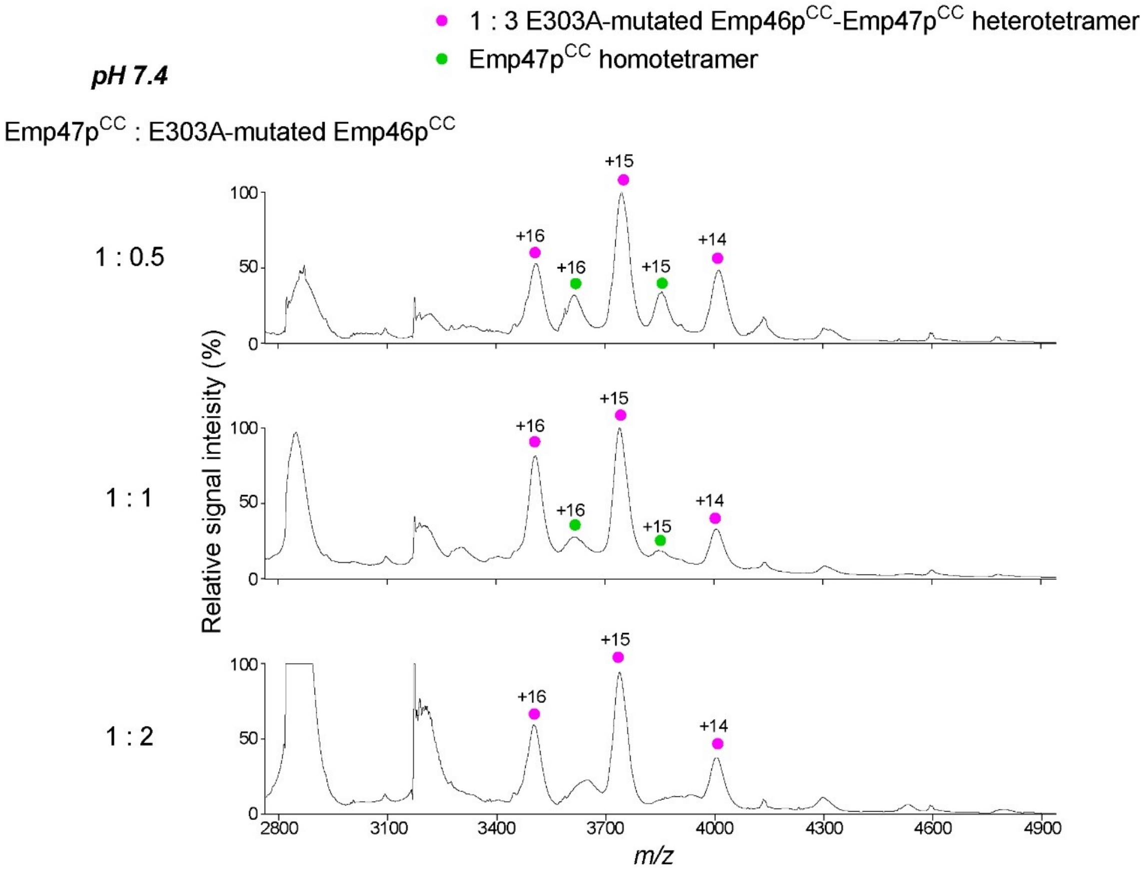

S6 Fig. Mass spectra of the mixtures of Emp47p^{CC} and the E303A-mutated Emp46p^{CC} at pH 7.4. The concentration of Emp47p^{CC} was fixed and that of the mutated Emp46p^{CC} was varied at the molar ratios indicated. Peaks corresponding to 1:3 heterotetramers of Emp47p^{CC} and the E303A-mutated Emp46p^{CC} are indicated by magenta dots with charge states. Peaks corresponding to the homotetramer of Emp47p^{CC} are indicated by a green dot with charge states.
